# Interventions to facilitate health workforce restructure

**DOI:** 10.1186/1743-8462-2-14

**Published:** 2005-06-29

**Authors:** SJ Duckett

**Affiliations:** 1 School of Public Health, La Trobe University, Melbourne Vic 3086, Australia

## Abstract

There are recognised shortages in most health professions in Australia. This is evidence that previous attempts at health workforce planning have failed. This paper argues that one reason for such failure is the lack of appropriate structures for health workforce planning. It also suggests that Australia needs to move beyond planning for particular professions and that health workforce planning needs to be based on identifying skill shortages as much as shortages in particular named professionals.

The paper proposes specific policy suggestions to facilitate workforce flexibility and health workforce planning in Australia.

## Background

Health workforce reform is clearly on the agenda of health policy makers in Australia. It has been the focus of discussion at the Council of Australian Governments which requested the Commonwealth to initiate further research in this area, operationalised by the Treasurer commissioning the current research study by the Productivity Commission . There are a number of immediate causative factors for this heightened policy attention, most notably contemporary perceived shortages of most categories of health professionals. Increasingly, health policy makers and health service managers are also recognising that the current structure of the health workforce is probably not suitable for 21^st ^century healthcare delivery. [1]

Australia is not unique in facing workforce shortages [2,3], nor in recognising the inadequacy of current workforce structures [4]; the World Health Organisation is highlighting workforce issues internationally by making them the focus of its 2006 World Health Report . Although the headline problem is usually couched in terms of workforce supply, problems in flexibility of the workforce and workforce planning also confront policymakers.

The focus on *workforce flexibility *is in part a response to perceived overspecialisation of the health workforce. Specialisation, which in part was seen to be associated with higher quality, is now seen as possibly detracting from continuity of care and hence may have a deleterious impact on quality, especially in the context of the increased salience of chronic diseases in the health sector. Although all the benefits of specialisation should not be lost, the current assignment of roles for health professionals is perceived to be inefficient either because more staff are employed than would be required in an efficient organisation of roles, or staff at higher pay classifications being used to perform tasks which could be performed by staff at lower pay levels. The inflexibility of contemporary workforce structure also inhibits service delivery because of shortages of staff to perform key roles. Policy attention is therefore being directed towards strategies about workforce substitution [5] and to develop skills "escalators", that is to make it easy for existing health professionals to acquire additional skills to enable them to perform additional tasks.

Table 1 shows some of the task substitutions which could potentially take place in Australia. In some cases the substitution is already occurring and the potential is for expansion of this practice. In other cases, substitution will require:

**Table 1:** Examples of potential (or current) task substitutions

Task*	**Traditional professional**	**Substitute professional/assistant**
Anaesthesia	Anaesthetist	Nurse anaesthetist
Clerking of new hospital patients	Hospital medical officer	Nurse
Closure of wound	Surgeon	Nurse
Foot care	Podiatrist	Foot care assistant
Foot surgery	Orthopaedic surgeon	Podiatric surgeon
Laryngoscopy/Naso-endoscopy	ENT surgeon	Speech pathologist/Nurse
Maternity care	Obstetrician	Midwife or GP
Mobilisation assistance	Physiotherapist	Physiotherapy assistant
Patient management	Medical practitioner	Nurse practitioner
Plain X-ray	Medical imaging technologist	X-ray assistant
Refraction	Optometrist	Orthoptist
Reporting pathology	Pathologist	Scientist
Reporting X-rays	Radiologist	Medical imaging technologist

* Performance of the substituted tasks will generally require additional training and clear protocols, and will also depend on the complexity of the condition and the comorbities of the patient

• Identification and clarification of the precise range of tasks to be substituted;

• Protocols to identify the types of patients for whom the substitute professional or assistant is relevant;

• Clarification of the nature of supervision, and reporting and regulatory arrangements (if any);

• Negotiation of payment/salary arrangements.

Obviously new substitution arrangements need to be carefully planned and monitored, but over time as health agencies (and patients) become more confident and familiar with substitution, expanded roles and task substitution will become a recognised and routine part of service delivery.

The possible substitution examples outlined above mostly involve changing the scope of practice of existing professionals. Substitution can also occur through creation of new categories of professionals or assistants. [6] The more prevalent substitution becomes, the more there will be challenges to our contemporary conception of the definition and place of a "nurse" or "physiotherapist". This will not be an issue for members of health care teams who work closely or regularly with team members working in extended roles, but transient team members (such as agency staff or staff with only irregular or peripheral contact with the team) may not be fully aware of the team's skill mix and may make inappropriate referrals or consultative decisions. Consumers may also have different expectations of the treating team membership, and this too will need to be addressed.

The importance of addressing workforce flexibility and the associated issue of workforce substitution cannot be underestimated, particularly as predictions of future workforce requirements need to make some assumptions about the mix of tasks that will be performed in the future by the health professionals under review [7,8]. If the tasks undertaken by physiotherapists, for example, are expanded, then more physiotherapists will be required, but if tasks currently undertaken by physiotherapists are able to be delegated to other categories of the health workforce, then the number of physiotherapists required in the future will be reduced. For this reason, the term 'skills shortage' is preferred to 'workforce shortage' to describe the contemporary problem. The latter term focuses on particular professions, thus channelling policy attention into traditional professional structures, rather than recognising workforce flexibility and the potential for changed skill mix.

**Figure 1:**
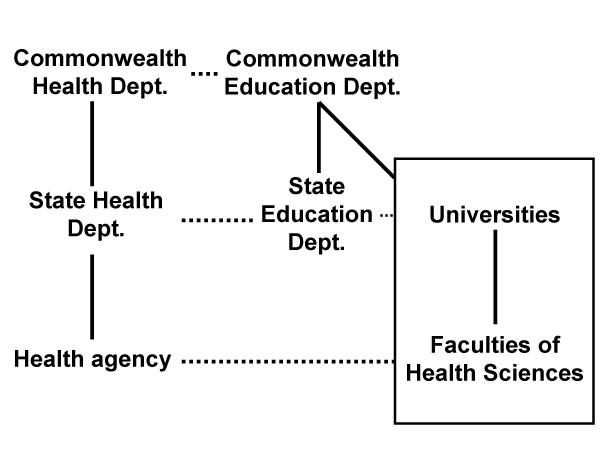
Organisational relationships between health and education sectors.

A second cluster of problems relates to health *workforce planning*. The legal aphorism, *res ipsa loquitur*, is relevant here. The existence of skills shortages damns current workforce planning efforts. Although there are technical problems with workforce and demand projections, a critical inhibiting factor is the lack of effective formal structural links between the health and education sectors. Figure 1 shows the current relationships.

A health agency, for example, relates most closely in organisational terms to the State Health Department. The State Health Department has an overview of the needs of the health agencies within a State and State Health Ministers may be politically exposed to shortages in particular health professions which lead to problems of service delivery.

State Health Departments have two sets of relationships which are of relevance here. One is to the Commonwealth Health Department structured through organisational arrangements such as the Australian Health Ministers' Conference and the Australian Health Ministers' Advisory Council. The other is to the parallel State Education Department. Relationships between state health and education departments are not always close and rarely involve structured joint planning arrangements. These somewhat looser relationships are indicated by dotted lines in the figure. The Commonwealth Health Department has links to the Commonwealth Education Department, which in turn has links to State Education Departments and to universities. At the bottom of the figure we note that health agencies have direct relationships with Faculties of Health Sciences within universities, for example, in terms of placement arrangements.

The mechanisms for a health agency or a State Health Department to influence the admission or curriculum decisions of universities are very indirect, typically progressing up and down the chain, mediated by the Commonwealth Departments. The longer the links in an implementation chain, the more the policies are likely to be attenuated or distorted. [9] The mechanisms for implementing health workforce decisions are very indirect and this could be predicted to be relatively ineffectual, which they are.

## Proposals for reform

The incentives on health services and universities are very different. The nature of accountability of the two sectors does not automatically guarantee that the two sectors would see the problems of the health workforce in a similar way, nor that they would accord the same priority to different solutions.

These differences, coupled with the indirect mechanisms of influence highlighted in the figure, mean that the perceived problems of the health workforce may not be easily resolved. Faculties of Health Sciences within universities are not autonomous, and even if they identify with the problems faced by the health sector, they may not have sufficient internal power within universities to effect change. University management may not recognise or accept a need to change university priorities or respond to the perceived problems of the health sector.

There are similar problems of alignment in terms of facilitating workforce flexibility. The structure of Australia's Medicare benefits arrangements militate against proposals for medical practitioners to promote substitution strategies.

These problems suggest that new strategies are necessary to change incentives to facilitate workforce reform; such strategies can be initiated by individual players in the health workforce policy area which would facilitate improvements in flexibility and in workforce planning.

## Workforce flexibility

In terms of workforce flexibility, one of the critical barriers to reform is the lack of financial incentives on the medical profession to engage in significant restructure of work roles. The Australian Medicare scheme places financial incentives on medical practitioners to provide services themselves because, in general, only services provided by the medical practitioner attract a rebate under the Medical Benefits Schedule. However, this is not universally true, and there are a number of items on the Schedule which do not require "personal provision" by the medical practitioner (see Section 12.1.1 of the Medicare Schedule, ). A typical item that does not require personal provision is a pathology test, where the test if performed by a medical scientist with the medical practitioner not necessarily being present, or even seeing the test result before it is issued.

One strategy to encourage flexibility in the workforce would be to increase the range of items which do not require personal provision by, for example, designating all procedural items in this category. In this way, for example, an anaesthetist would be able to bill for the work of a nurse anaesthetist using the anaesthetic items of the Schedule. Assuming salary costs for the substitute professional are lower than the medical specialist, this would then put a financial incentive on medical practitioners to utilise other health professionals for service delivery. It may also be appropriate to allow some consultation items to be billed without personal provision, eg. if provided by a nurse practitioner/advanced practice nurse. This change could be undertaken by the Commonwealth Department of Health unilaterally. A change to the 'personal provision' rules of the Schedule can be undertaken without affecting contemporary fee relativities. However, procedural items are often claimed to be over valued relative to non-procedural items, and a change in the personal provision rules could also involve some realignment of the Schedule to facilitate a greater recognition of the cognitively complex components of patient care which will remain the preserve of medically qualified practitioners.

Medical practitioners are rightly concerned at their place in the health care system. The political voices of the medical profession thus generally oppose structural changes which reduce medical autonomy or might increase competition and impact on medical incomes. A change to the Medicare Benefits Schedule of the kind proposed would ameliorate these concerns and might thus enable the medical professional to support such changes without any threat to their roles and income. It should be noted, however, that delegation from medical practitioners is not and should not be the only possible source of income to support new or expanded roles.

A parallel change could be to introduce powers of delegation within health professional registration Acts. A power of delegation would facilitate professionals delegating tasks by extending the reach of a health professional registration board to cover the work of any person to whom a professional registered with that board has delegated tasks (see, for example, . [10] Such a power of delegation would establish a regulatory framework for health professionals' delegating to other professionals or assistants, and would allow professionals to delegate tasks, knowing they were doing so within an accepted regulatory framework.

Thirdly, the Medical Benefits and Pharmaceutical Benefits arrangements (and associated state regulatory controls) could be changed to give other professionals access to test ordering or prescribing authority. This is already occurring through expanding nurse practitioner access to MBS and PBS arrangements, but it could also apply to podiatrists, optometrists, physiotherapists and other health professionals.

These flexibility arrangements should be accompanied by parallel educational reforms to facilitate upskilling and reskilling of health professionals. In this regard, increased graduate entry programs for health professionals, whereby graduates from other disciplines are able to undertake shortened courses to gain professional recognition, should be encouraged. Shortened courses for professionals to acquire some of the key skills beyond their normal range should also be developed (eg. nurses to be trained in foot care). Similarly, universities should be encouraged to provide modular and multiple pathways for developing health professionals. For example, there should be expanded arrangements for dental therapists to upgrade to become dentists. Similarly, there should be multiple pathways for training of psychiatric nurses, providing structured courses for people with an initial nursing background and for people with an initial psychology background.

Not all skill upgrades will need to involve university programs. Health agencies (and professional registration bodies) may cooperate to develop work-based programs to address skill gaps. Such programs should be conducted with a recognised qualification framework to ensure portability. The Vocational Education and Training sector may have a role here, possibly in collaboration with universities.

## Changes to structures

The changes to facilitate flexibility outlined above can be undertaken unilaterally by States changing registration Board legislation or by the Commonwealth changing the MBS arrangements. However, in the long term there needs to be reform to funding and management structures to improve health workforce planning in Australia. The Nelson changes to higher education have improved the accountability of universities though closer monitoring of the course mix within universities. But this monitoring is still undertaken at a very broad level and cannot be expected to go into the detail of particular health professions. Further, previous experience suggests that, although the Department of Education will have an initial flush of enthusiasm for close monitoring, this enthusiasm abates and the relationships between the Commonwealth Department of Education and universities become more *laissez faire *over time. [10]

The situation in Australia for funding universities for health professional education contrasts with that in the United Kingdom where, for most health professions, there is a direct contractual relationship between the health sector and universities. For example, although degree-level education for nursing is funded through standard education sector funding for universities, diploma-level training, which represents the overwhelming majority of university-based nursing education in the United Kingdom, is funded in universities through a direct contract between the university and the National Health Service. This helps to ensure that universities are more accountable to the health sector to provide relevant and appropriate health professional education graduating adequate numbers of professionals. Such contracts are comprehensive, providing for an ongoing relationship between the health service and the university, and are not simply based on selection of the cheapest provider. It also provides a framework for experimentation and responsiveness in terms of preparing new types of health workers. The potential of these arrangements has not been fully realised, although there are pockets of innovation (see ). In the early years of NHS purchasing of professional education, the NHS tended to underestimate demand for health professionals from non-NHS agencies [7], highlighting the need for comprehensive skills planning.

There are lessons for Australia here, and more direct links between the health sector and universities could improve responsiveness of universities to emerging needs. The first stage of such a closer relationship could occur if the Commonwealth assigned responsibility for health professional education to the Department of Health and Ageing rather than the Department of Education, Science and Technology. Universities already face multiple sources of funding, and a shift of responsibility for health professional education to the Department of Health and Ageing would give that Department a direct involvement in setting priorities for the future health workforce and funding universities accordingly. In contrast to the Department of Education, Science and Training, the Department of Health and Ageing is much more likely to have an ongoing and continuing interest in ensuring adequate numbers of health professionals and the competencies attained by new graduates. Similarly, many reports commissioned by the Department of Health lament the adequacy of the curriculum of universities in a range of areas, but there have been few levers over universities to effect relevant changes. A shift of responsibility would reduce the number of links in the chain between health agencies and universities in terms of responsiveness and skills planning. Such a change in funding source could be undertaken unilaterally by the Commonwealth Government.

State health authorities could also exercise influence over universities. In many States, the State health authority provides subsidies to universities either directly or indirectly for professorial appointments. Many clinical Chairs in medicine, nursing and other disciplines, are funded by the State health authority (or health agencies). Similarly, health professional education would not be viable were it not for the access to State-funded health agencies for clinical education. Controlling this access could thus give State health authorities some levers over universities. Given these levers, State health authorities could take a much more direct role in negotiating with universities about health professional education than they have hitherto. This change could be undertaken unilaterally by any State, regardless of any changes in Commonwealth responsibilities.

Finally, it would be better if the actions of the Commonwealth and the States were brought together into a coherent policy approach. This could be done informally through arrangements for joint Commonwealth/State negotiations with universities. A stronger policy could be to establish a single funding pool to which both the Commonwealth and the States contribute, which would facilitate direct negotiations between the Commonwealth and State Governments on one hand, and universities on the other. A single funding pool could be administered by a jointly established health workforce funding agency in each State which would have the full purchasing responsibility for health professional education. These arrangements would, of course, be more complex to implement but would be much more powerful mechanisms for reform. Coordination of state and Commonwealth activity would also facilitate engagement with the private sector. Given the different roles of the public and private sectors in health delivery, it is important to develop structures to engage the private sector more directly in educating the future health workforce which will be employed in that sector. Involvement of the private sector in the skills planning process would also help to ensure that demand from that sector is taken into account in supply decisions, overcoming one of the early weaknesses experienced in the similar arrangements in the United Kingdom.

Universities might benefit from more systematic planning arrangements for health professional education. At present universities often have problems negotiating clinical education arrangements with health agencies, where there are few incentives on agencies to assume responsibility for education of the next generation of health professionals. A *quid pro quo *for increased university accountability to State or Commonwealth health departments would be an increased responsibility on either the Commonwealth or the State governments (or both) to ensure the adequacy of clinical education arrangements for universities. A direct relationship would also make it more likely that skills upgrading would remain within the purview of universities rather than bypassing them. Finally, distinct education funding arrangements for the health sector would mean that government could allocate more funding per health student without creating a precedent for increases in other disciplines.

## Conclusion

There are a number of contemporary problems of health professional education. Many of these problems have been identified for decades, but there have been few incentives to achieve change and/or the structural mechanisms for change have hitherto not been present. Past opportunities have been missed, eg. in the negotiation of the 2003–2008 Australian Health Care Agreement. The current heightened policy awareness of the need for workforce reform provides a new opportunity for change. Discussion, planning and experimentation should commence now to provide a sounder conceptual and evidence base to ensure that opportunities are not missed to incorporate reform proposals in the 2008–2013 Australian Health Care Agreement.

In this paper I have outlined incremental steps that could be used to facilitate change in health workforce policy in Australia. The general tenor of the changes are that they provide for increased accountability of universities. But the costs of these changes do not fall only on universities. Introducing new mechanisms to hold universities accountable for adequacy of health professional education in turn means that governments themselves are more clearly accountable for the adequacy of health professional education, and shortages in any discipline would be more clearly seen to be as a result of government decisions. Governments (both Commonwealth and State) benefit from the ability to blame shift to other participants in the health workforce policy arena.

New structures for the health workforce and for health workforce planning are clearly necessary in Australia. In this paper I have outlined a possible win/win scenario for policy reform to address these needs.
